# Acute and Chronic Hyponatremia

**DOI:** 10.3389/fmed.2021.693738

**Published:** 2021-08-03

**Authors:** Murad Kheetan, Iheanyichukwu Ogu, Joseph I. Shapiro, Zeid J. Khitan

**Affiliations:** Department of Internal Medicine, The Joan C. Edwards School of Medicine, Marshall University, Huntington, WV, United States

**Keywords:** hyponatremia, osmolyte, syndrome of inappropriate antidiuresis, overcorrection, myelinolysis, central pontine

## Abstract

Hyponatremia is the most common electrolyte disorder in clinical practice. Catastrophic complications can occur from severe acute hyponatremia and from inappropriate management of acute and chronic hyponatremia. It is essential to define the hypotonic state associated with hyponatremia in order to plan therapy. Understanding cerebral defense mechanisms to hyponatremia are key factors to its manifestations and classification and subsequently to its management. Hypotonic hyponatremia is differentiated on the basis of urine osmolality, urine electrolytes and volume status and its treatment is decided based on chronicity and the presence or absence of central nervous (CNS) symptoms. Proper knowledge of sodium and water homeostasis is essential in individualizing therapeutic plans and avoid iatrogenic complications while managing this disorder.

## Introduction

Hypotonic hyponatremia is the most common electrolyte disorder encountered in clinical practice (1). In most cases, it is the result of impaired free water excretion due to the inability to suppress antidiuretic hormone (ADH). It can also result from polydipsia when water intake overwhelms the maximum renal diluting capacity. In this review, we are discussing the role of Neuronal adaptive responses to the hypotonic state that accompanies hypoosmolar hyponatremia focusing on the role played by the organic osmolytes. We are also reviewing the pathogenesis and manifestations of hyponatremia as well as treatment options and guidelines. While managing patients with hyponatremia, it is very important to keep in mind their risks for complications from the acute state as well as risks of demyelination in the chronic state. This should aid in individualizing treatment plans and avoid iatrogenic complications.

## Organic Osmolytes

Central to the clinical topic of acute and chronic hyponatremia is the subject of organic osmolytes. Although these chemicals in the brain were once referred to as “idiogenic osmoles,” they have been extensively characterized and measured (2). Although any non-electrolytic compound exerts an osmotic force, the amount of said osmotic force is directly proportional to the concentration or activity of the chemical in the solution being studied. In the context of brain organic osmoles, the major chemical classes for these are the methylamines such as glycerolphosphorylcholine and betaine, amino acids such as taurine, glutamine and glutamate and polyols such as sorbitol (2–4). Urea is an organic, osmotically active molecule, but as it diffuses rather quickly through lipid bilayers, it is not felt to be an “effective” osmolyte. Also, as the other classes of osmolytes are believed to stabilize protein quaternary structures, urea is known to denature proteins (5). Features of organic osmolytes are summarized in Table 1.

**Table 1:** Features of organic osmolytes.

**Chemical clas**	**Examples**	**Rapidity of regulation**
Polyols	Sorbitol, myoinositol	Medium
Amino acids	Taurine, proline, glutamate	Rapid
Methylamines	Betaine, Glycerophosporylcholine	Slow
MIsc	Urea	Very rapid

It is important to understand why we have such osmolytes in the first place. Single cell organisms have systems to regulate the intracellular concentrations of these chemicals. At the risk of oversimplification, it appears that concentrating organic osmolytes allows not only for the stabilization of cell volume when external ionic strength is high but also for the stabilization of protein structure and function under these conditions (6).

There is a time component to the regulation of the intracellular concentrations of organic osmolytes. In the mammalian brain, the first defense against brain swelling with acute hyponatremia is believed to involve a hydrostatic shift in fluid from brain to cerebrospinal fluid (CSF) and ultimately to the systemic circulation (7, 8). The second involves the active depletion of ions within brain cells. Specifically, decreases in the intracellular concentrations of potassium, sodium and chloride occur quickly with a new steady state reached after about 3 h (9).There appears to be a limit to this mechanism of adaptation, and it is clear that brain swelling is the predominant form of brain injury from acute hyponatremia (10). The increased risk of adverse outcomes in menstruant females reported by Arieff and colleagues appears to be associated with swelling at a cellular level (11–14).

**Figure 1:**
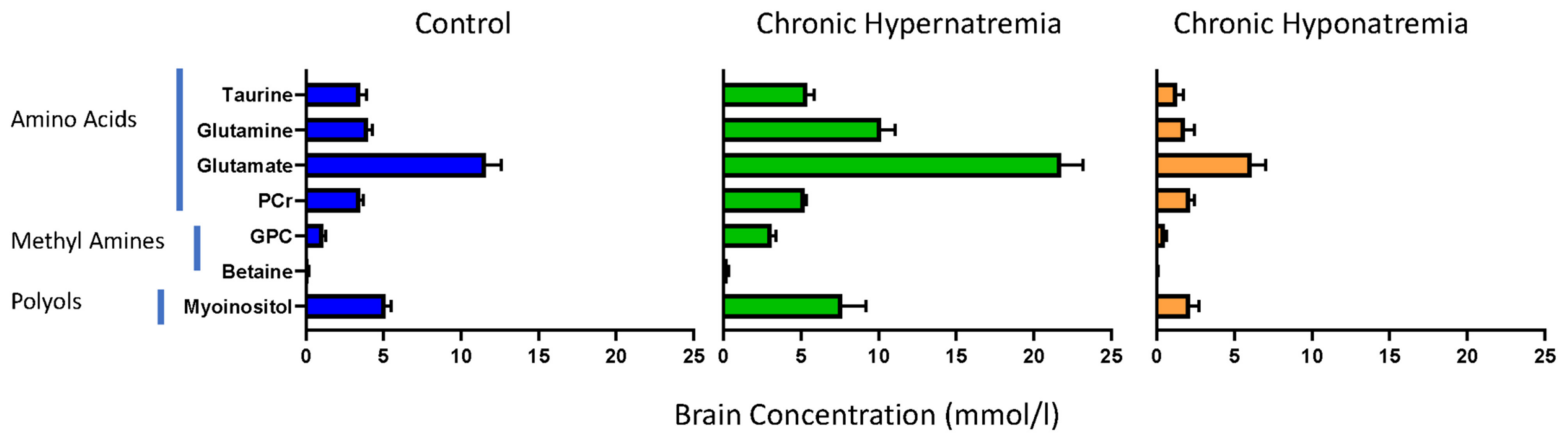
Brain organic osmolytes by chemical class in normal rats (left, *N* = 8) and rats exposed to chronic hyponatremia (right, *N* = 6) and hypernatremia (middle, *N* = 6). From references (4, 16).

In contrast, the slow development of hyponatremia, even to very severe degrees, is not complicated by significant brain swelling (15). This is probably related to the regulation of brain organic osmolytes which is, as mentioned earlier, time dependent. However, the time involved in regulation of organic ion concentrations is not uniform. Changes in amino acid concentrations appear to occur the quickest with polyols next and methyl amines, in particular, glycerophosphorylcholine, slowest (4, 16). Levels of different osmolytes in different states of chronic dysnatremias are shown in Figure 1. Important to note that there are also brain topographic differences in the timing of organic osmolyte regulation. The temporal dissociation between organic osmolytes concentration in brain tissues and the presence of demyelinating lesions with treatment, which represent the hallmark of treatment complications, has been well-established (17). In humans, the region of brain at greatest risk appears to be the pons (18), whereas in experimental rodent models the midbrain appears to be at greatest risk (17). It is important to note that clinical demyelination syndromes following rapid correction of hyponatremia in humans are not limited to the pons (19).

### Brain Injury With Acute Hyponatremia

Hypotonic hyponatremia primarily presents with symptoms of CNS dysfunction. These symptoms can range from mild, as in impaired taste sensation, muscle cramps, and nausea to moderate as in weakness, confusion, and delirium, to severe, in which altered mentation and seizures can occur. The severity of these symptoms depends on the etiology, the size of the drop in sodium concentration and the acuity or rapidity of that drop. These symptoms are more pronounced and can be life threatening when the drop in serum sodium concentration is large, <120 mmol/L, and acute, i.e., within few h (20).

Acute hyponatremia commonly occurs when the amount of water intake exceeds the ability to excrete electrolyte-free water by the kidneys, whether the renal water excretion is impaired due to inappropriate or non-osmotic release of ADH, or the excretion mechanisms are overwhelmed by large quantities of water. Some of these cases are seen in acute water intoxication and marathon runners (large amounts of water intake) (21) or *in situations* when there is inappropriate release of ADH that is primarily triggered by a nonosmotic stimulus as in stress in the post-operative state, HIV, CNS injury, pulmonary pathology and tumors (22–25).

Neuronal cellular response to the hypoosmolar state is a key determinant of encephalopathy that is affected by the severity and acuity of hyponatremia. The brain capillaries, unlike systemic capillaries, have tight endothelial junctions that are part of a neurovascular unit composed of astrocytes, with their foot processes lining the capillaries, pericytes and extracellular basement membrane forming the blood-brain barrier (BBB) (26, 27). Unlike systemic capillaries, were sodium readily crosses the intravascular compartment to the interstitial compartment, the BBB limits the entry of hydrophilic substances.

As part of the adaptive response of the brain to the hypoosmolar state that is associated with hyponatremia, water will move into the brain tissue, along the osmotic gradient, through the aquaporin-4 channels expressed on the foot processes of the astrocytes, in an attempt to limit osmotic stress injury to the neurons (28). This will result in swelling of the brain cells and brain edema. The brain adapts to the increase in volume by shunting the water through the astrocytes, while avoiding the neurons (29) and by losing intracellular and extracellular osmolytes in a process called volume regulatory decrease (VRD) (28). Astrocytes utilize energy dependent mechanisms that require the Na^+^-K^+^-ATPase system to expel K^+^ and Cl^−^ while organic osmolytes like glutamate, glycine, taurine, creatine, myoinositol, and GABA are translocated through leak-pathways, hence decreasing volume and edema (30, 31). Of note, organic osmolytes, e.g., glutamate and GABA, have a dual function of being neurotransmitters in addition to being osmolytes; loss of glutamate from CNS cells can predispose to seizures, which is one of the clinical presentations of hyponatremic encephalopathy (32).

In addition to the acuity and the size of the drop in serum sodium, the severity of hyponatremic encephalopathy and mortality seem to be affected by the gender of the patient. In 1986, a case series reported 15 previously healthy female patients with a mean age of 41 years who developed severe hyponatremia about 49 h following elective surgery. Subsequently all of them either died or had permanent brain damage (14). A case-control study in 1992 found that post-operative hyponatremic encephalopathy is equally likely in both genders but women of menstruating age were 25 times more likely to die or develop permanent brain damage than either men or postmenopausal women (33). In endurance and ultra-endurance sports, like marathon and triathlon competitions, exercise-associated hyponatremia (EAH) was more prevalent and more severe with worse outcomes in women than men (21, 34). In 2005, a study, which looked at hyponatremia among runners in the Boston Marathon, found that female sex was not associated with hyponatremia, while extremes of body-mass index (BMI), weight gain while running and long racing time were associated with hyponatremia (35).

There are multiple proposed mechanisms that have been linked to the higher incidence of hyponatremia and hyponatremic encephalopathy in women. Female sex hormones have been shown to inhibit the activity of the Na^+^-K^+^-ATPase system, since estrogens share a similar core steroidal structure with ouabain and cardiac glycosides (known Na^+^-K^+^-ATPase system inhibitors), hence impairing the astrocyte volume regulation (36, 37). In an animal study, estrogen appeared to alter water movement and neurotransmission in the hippocampus by affecting aquaporin-4 expression (36). Finally, interleukin-6 (IL-6), which is released from skeletal muscle injury and is found at a higher level in women than men (37), is assumed to play a role in vasopressin secretion and reduce the expression of aquaporin-2, hence impairing free water excretion (38).

Therefore, acute symptomatic hyponatremia is considered an emergency that carries a high risk of mortality and morbidity (39). Immediate action is necessary to reverse the osmolar injury and brain edema.

### Chronic Hyponatremia

In chronic hyponatremia (>48 h), the losses of both electrolytes and organic osmolytes (e.g., myoinositol, betaine, glutamine, taurine, and g-aminobutyric acid etc.) from brain cells are efficient mechanisms that regulate brain volume and thus minimize brain swelling and neurological symptoms (40, 41).

Mild or moderate hyponatremia had been previously referred to as asymptomatic hyponatremia (42). Unfortunately, this condition is not completely benign and can be associated with some subtle complications including attention deficit, lethargy, restlessness, disorientation, headache, nausea and vomiting, muscle cramps, falls, gait abnormalities and depressed neural reflexes, some of which are reversible upon improvement in serum sodium concentration (43, 44).

Several of the organic osmolytes lost from brain cells in the adaptive process of hyponatremia, in particular glutamate, are neuroactive and therefore could produce neurological abnormalities such as decreased synaptic release of excitatory neurotransmitters, which could explain the gait instability observed in chronically hyponatremic patients (40, 41, 45). In a study where attention tests were administered to patients with moderate hyponatremia and normal control subjects, hyponatremic patients had significantly higher error rates and more gait disturbances (46). Moreover, chronic hyponatremia is associated with increased risks of falls and injurious fractures in the elderly (47).

Fractures in patients with chronic hyponatremia are thought to arise from falls resulting from mild cognitive impairment and unsteady gait and directly from osteoporosis and increased bone fragility (48). Adults with mild hyponatremia have been shown to have a significantly increased risk for osteoporosis at the hip and femoral neck. These observations may be related to stimulation of osteoclastic activity and enhanced bone resorption in the setting of a low serum sodium concentration (49).

In primary care setting, all levels of hyponatremia are associated with all-cause mortality (50). Hyponatremia is a major risk factor for mortality in advanced liver cirrhosis and has been noted to be associated with increased mortality, rehospitalizations, prolonged hospital stays, and major cardiovascular events in patients with heart failure (51). It is also associated with higher risk for all-cause mortality in non-dialysis CKD and maintenance dialysis patients (52).

### Consequences of Overly Rapid Correction

As previously stated, since chronic hyponatremia develops slowly, it allows the brain to compensate considerably by cellular exit of electrolytes and organic solutes that promotes water loss thus ameliorating brain swelling and minimizing symptoms (53). This adaptive process in chronic hyponatremia predisposes the brain to the development of osmotic demyelination syndrome (ODS) in the event serum sodium is rapidly corrected while re-accumulation of organic osmolytes is delayed. ODS occurs especially in the pons (central pontine myelinolysis), although extrapontine myelinolysis affecting the basal ganglia, cortex, lateral geniculate body and internal capsule can also occur (40, 41).

Transfer of taurine to adjacent astrocytes protects the neurons from osmotic stress and allows them to maintain their volume. Within 24–48 h after this transfer, astrocytes restore their volume through loss of organic osmolytes and down regulation of transporters (54). However, in the setting of a hypertonic stress due to rapid correction of hyponatremia the astrocytes are unable to rapidly reaccumulate depleted osmolytes, which then leads to disruption of the BBB (53, 54). In rats, rapid correction of chronic hyponatremia leads to rapid rises in brain Na^+^ and Cl^−^ to supernormal levels while it takes several days for the organic osmolytes to reaccumulate in brain cells (16, 55). The variability of organic osmolyte reuptake by brain cells in different states can alter the risks of ODS. Reuptake of the myoinositol and other organic osmolytes occur more rapidly in the uremic environment which explains the low risk of ODS in uremic subjects (56). Astrocyte apoptosis is followed by the loss of the communication between astrocytes and oligodendrocytes, which is crucial for the myelination processes (40, 41). Following astrocyte death, it is predicted that inflammation induced by pro-inflammatory cytokines and microglia activation along with complement activation following the disruption of the BBB eventually result in demyelination (40, 41, 57).

Clinically, ODS manifestations may include quadriparesis, dysarthria, dysphagia, and other pseudobulbar symptoms, pseudobulbar palsy, seizures, locked-in syndrome, coma and even death (58). Usually, the development of these symptoms may occur several days after the correction of hyponatremia and in some cases, as suggested from autopsy series, ODS may be asymptomatic or mildly symptomatic (40, 41). These symptoms may or may not be reversible (58).

### Treatment of Hyponatremia

Treatment of hyponatremia relies on the understanding of the CNS adaptation to altered serum osmolality and on the risks of complications from hyponatremia and its correction. The following factors that can affect the outcome of treatment should be considered while individualizing the therapeutic plan: (1) Severity of hyponatremia as determined by serum sodium concentration, (2) symptoms of altered central nervous function such as delirium, seizure, or coma and (3) acuity of hyponatremia (<48 vs. >48 h).

### Using Formulas to Guide Therapy and Predict Correction Rate

The challenge in treating hyponatremia is to predict the rate of correction by each individual therapy in a specific patient. This requires individualized knowledge and understanding of the mechanisms of hyponatremia aiming for an appropriate rate of correction necessary to prevent unwanted consequences. Several formulas have been utilized in this regard to aid physicians in planning the therapeutic approach for their patients. Given their theoretical limitations, and the made assumptions, these formulas should guide therapy rather than replace frequent serum sodium concentration monitoring.

Quantification of free water excess (FWE) (59) can be an attractive way of planning and predicting the rate of correction of hyponatremia. Knowing that total body water (TBW) is roughly 50–60% of total body weight with the lower limit in females due to higher fat content, one can calculate FWE by the following formula:

$$\begin{array}{l} FWE=TBW\;\times \;\frac{140-Serum\;[Na^{+}]}{140} \end{array}$$

By defining the desired sodium concentration, one can decide on the amount of free water that needs to be excreted over the next 24 h. This amount can be achieved by using loop diuretics as a method to downgrade urine concentration which can dilute the urine and simultaneously replacing sodium according to urine sodium and potassium excretion rates. In this calculation, it is assumed that the patient is euvolemic and that serum sodium is the only determinant of serum osmolality.

A different approach is to estimate the direct effect of a liter of a given fluid on serum sodium concentration [S (Na^+^)] which can be predicted according to the following formula (60):

$$\begin{array}{l} Increase\;in\;S[Na^{+}]=\frac{\left(Infusate\;\left[Na^{+}+K^{+}\right]-S\left[Na^{+}\right]\right)}{TBW+1} \end{array}$$

Although it is widely used, this formula assumes that the human body is a closed system without paying attention to the urine composition. Moreover, it ignores the effect of volume management in hypovolemia which potentially can lead to overcorrection of hyponatremia due to the attenuation of the hypovolemic stimulus on ADH release.

Another approach validated by Edelman et al. (61) and simplified by Sterns (54) focused on body water-two ion model, where body fluids can be considered as being in a single tub (after eliminating cell membranes) containing Na^+^ and K^+^, the most prominent extracellular and intracellular cations, respectively, and water. When applying this concept [Na^+^], is altered by the net external balances of Na^+^, K^+^ and water as represented in the equation:

$$\begin{array}{l} \left[Na^{+}\right]=\frac{Na^{+}+K^{+}}{H2O} \end{array}$$

**Figure 2:**
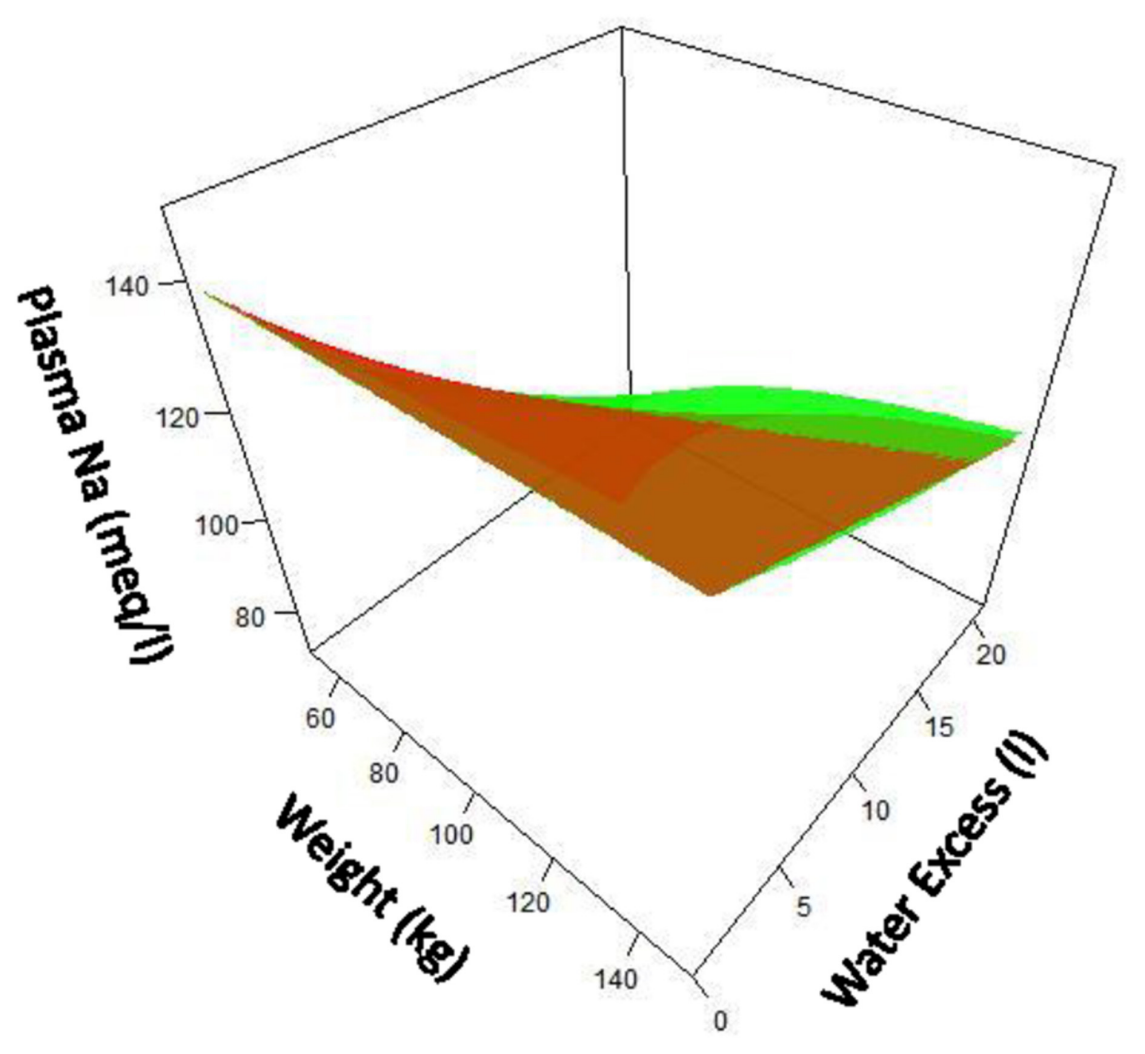
Comparison of “simple” formula (green) for serum sodium compared with Edelman formula (red) over ranges of patient size and water excess. Assumption made that fraction of water is 0.6 X body weight. Note that at lower patient size and greater water excess, difference between two formula becomes notable.

Where Na^+^, K^+^, and H_2_O are, respectively, total body rapidly exchangeable sodium, total body exchangeable potassium, and total body water. Using this equation, [Na^+^] can be predicted by the net change in Na^+^, K^+^ and water content assessed by their intake and their concurrent urinary losses. Figure 2 compares the “simple” and Edelman's formulas.

### Strategies Used in the Therapy of Hyponatremia

#### 1-Water Restriction

Water restriction is indicated for the treatment of all hyponatremias in the early phases particularly when associated with fluid overload states, the syndrome of inappropriate antidiuretic hormone secretion (SIADH) and advanced renal failure (60). In order to be effective, fluid intake has to be less than urine output and the sum of urine [Na^+^] and [K^+^] to serum [Na^+^] ratio is <0.5 (62) otherwise loop diuretics can be considered to achieve more effective urine dilution. Extreme fluid restriction (<800 cc) if needed can be difficult to achieve and other measures may need to be considered such as the infusion of hypertonic saline with or without loop diuretics. In this case, it is important to stress that hypertonic saline in addition to a loop diuretic is an aggressive maneuver that should be reserved for life-threatening hyponatremia.

#### 2-Urea and Salt Supplementation

Urea has a unique property that makes it an attractive agent to treat hyponatremia. Urea is an ineffective osmole due to urea transporters that facilitate urea diffusion across most cell membranes but has a reflection coefficient of 0.5 across brain capillaries (63). Therefore, administering urea to hyponatremic patients results in rapid resolution of brain edema by its osmotic property across the BBB along with the induction of electrolyte-free water diuresis. Thus, as the urea gradient across the BBB dissipates, it is replaced by an increase in serum sodium concentration that prevents plasma water from reentering the brain (63).

Oral sodium chloride replacement along with fluid restriction with or without a loop diuretic is a common practice used to treated SIADH in the outpatient setting. The relatively constant urine osmolality along with the normal renal sodium handling in SIADH can result in the increase in urine output. The usual dose of sodium chloride used by many practitioners (3 gm daily) is likely insufficient and has been shown to have no advantage over water restriction alone (64). Therefore, a higher dose (4–6 gm daily) with loop diuretic given on twice or thrice a day schedule is likely to be more effective.

#### 3-Saline and Hypertonic Saline

Fluids with different tonicity can be used to treat different types of hyponatremia. The choice of fluid depends on the etiology of hyponatremia as well as its severity and the associated symptoms. Isotonic saline is generally used to treat hypovolemic hyponatremia. Certain cases of severe hyponatremia (serum sodium <120 mEq/l) may require use of hypotonic fluid at some point. Restoration of euvolemia leads to suppression of the non-osmotic vasopressin release thus increasing the chance of overcorrection. This can be identified by monitoring urine osmolality. Hypotonic fluids may be also needed for certain conditions associated with low serum vasopressin levels and dilute urine, such as beer potomania and primary polydipsia. The use of isotonic saline is discouraged in symptomatic isovolemic hyponatremia secondary to SIADH while hypertonic saline (3%) is used instead. Loop diuretics can be added in certain scenarios when decreasing urine osmolality is required.

#### 4- Hypertonic Saline and Desmopressin

Over the past decade, the concurrent administration of hypertonic (3%) saline infusion and serial dosing of desmopressin over 48 h period have gained popularity and became the preferred method of treating severe hyponatremia in many centers. In this approach, hypertonic saline is infused [infusion rate calculated according to the desired [Na^+^] (60)] to ensure prompt correction of hyponatremia while desmopressin is given to prevent rapid free-water diuresis and the chance of overcorrection should the cause of hyponatremia is removed (65). This method allowed physicians to take control while managing severe hyponatremia especially in certain situations when urine osmolality is expected to rapidly decline predisposing to overcorrection of [Na^+^]. This approach can be effective in certain conditions when rapid correction of [Na^+^] is predicted as in hypovolemic hyponatremia and drug induced hyponatremia when urine osmolality can rapidly decline following volume repletion in the former and fading drug effect in the latter (66).

#### 5-Vaptans

The polypeptide vasopressin antagonist was first synthesized in the late eighties of the preceding century (67) but was far from being ready for clinical use due to its poor bioavailability and residual agonistic activity (68). More than 15 years later, conivaptan then tolvaptan were granted the FDA approval to treat euvolemic hyponatremia in the US. Initially, there was a great deal of enthusiasm toward these medications as they were targeting the very mechanism leading to euvolemic hyponatremia but this enthusiasm had dissipated in the following years as a result of several factors including: Too slow to benefit patients with hyponatremia who have severe cerebral symptoms, unfavorable side effects profile, lack of a measurable benefit when compared with alternative treatments of hyponatremia and finally cost (68).

## Treatment Recommendations

The complex treatment of true hyponatremia relies on preventing cerebral edema and herniation in the acute setting and avoiding the iatrogenic complications that can result from unnecessary treatment or inappropriate correction rate in the chronic cases. Considering the acuity and severity of hyponatremia, clinical manifestations including the existence of severe symptoms like seizure, risk factors of cerebral edema and risks of over-correction as well as risks of ODS are crucial in individualizing the management plan.

In acute hyponatremia, cerebral edema and brain herniation are the most serious clinical manifestations (69, 70). Nonspecific symptoms like nausea, vomiting and headache can rapidly progress to seizure and respiratory arrest. Early detection and rapid management are key factors in preventing this dreaded complication.

Limited literature concluded that 4–6 meq/L increase in serum [Na^+^] is sufficient to manage brain edema in patients with acute hyponatremia (71). This was supported by earlier literature showing that increasing serum [Na^+^] in normonatremic patients with cerebral edema by 4–6 meq/L using hypertonic saline resulted in significant decrease in intracranial pressure and reversal of transtentorial herniation (72). Similarly, brain edema in marathon runners can safely be treated in the field with hypertonic saline administered as 100 ml dose that can be repeated in resistant cases prior to hospital transfer (73).

The United States guidelines recommended the use of hypertonic saline in patients with acute (<48 h) hyponatremia with moderate to severe symptoms (74) while the European guidelines based its recommendations to use hypertonic saline on the severity of symptoms rather than the duration (75). It is important to point out that the European guidelines recognized vomiting to be a severe symptom along with respiratory arrest, seizure, somnolence, and coma. Both guidelines agreed to use hypertonic saline boluses with comparable doses ranging between 100 and 150 ml over 10–20 min that can be repeated 2–3 times until resolution of the symptoms (74, 75). This approach is expected to increase serum [Na^+^] by 4–6 meq/L resulting in the reversal of the cerebral edema. The rate of correction after the initial administration of hypertonic saline need not to be restricted when there is certainty of true acute hyponatremia nor is relowering of the serum sodium concentration required in case of excessive correction (74). However, if there is any uncertainty as to whether the hyponatremia is chronic or acute, the limits for correction of chronic hyponatremia need to be followed as shown below (74). A recent study found both the slow continuous infusion and the rapid intermittent boluses of hypertonic saline in symptomatic hyponatremia patients to be effective and safe with no difference in the overcorrection rate with essentially the same results relative to the rate of correction and resolution of symptoms (76). Therefore, hypertonic saline boluses can be the preferable regimen especially when rapid partial correction is desired in patients with cerebral edema as they limit calculations using correction formulas and chances of overcorrection (77).

Over the preceding few decades, variable recommendations on the rate of correction of chronic hyponatremia were made. The rapid rate of correction was clearly denounced as being the cause of permanent neurological sequalae but recommendations for limits of the increase in [Na^+^] were variable. In most studies linking the rate of correction of chronic hyponatremia to outcomes, the hourly rate of correction was predicted by equal distribution of the total rise in serum [Na^+^] over the 48 h duration (74). This rate can be misleading particularly if treatment upon diagnosis was delayed which makes the true rate of rise in serum [Na^+^] higher than reported (<48 h) or if the treatment was extended for several days with very low starting point accompanied by an uneven hourly rate of correction (74). When the rate of correction is higher in the second day of treatment, the uneven hourly rate of correction will falsely be reported as a relatively low rate of correction although the bulk of serum [Na^+^] correction happened later in the treatment course at a time when chronicity is more noticeable and carries a higher risk of neurological sequelae (78).

The current guidelines focus on risks of ODS when defining correction rate of chronic hyponatremia (74). In patients at higher risks of ODS [serum [Na^+^] <105, hypokalemia, alcoholism, advanced liver disease and malnutrition] (74), a minimum daily correction rate of 4–6 meq/L was recommended vs. 4–8 meq/L for low risk patients with a limit of correction set at 8 meq/L and 10–12 meq/L, respectively. This is based on the safety of this rate of correction that was shown in a large study (79). Although lower rates (<4 meq/l) can be associated with excess mortality (80), there is no evidence that exceeding the current recommendation has any benefit.

Overly rapid correction of chronic hyponatremia (>10 meq/L during the first 24 h and >8 meq/L every 24 h thereafter) can have serious consequences if ODS develops (75). It is likely to occur in subjects with rapid restoration in renal diluting capacity as in managing hyponatremia in the setting of volume depletion or drug-induced SIADH. There is lack of randomized controlled trials addressing management of rapid correction of hyponatremia but experts suggested intervention by administering electrolyte free water or injecting desmopressin to re-lower [Na^+^] (75). A recent large retrospective analysis reported 41% rate of rapid correction of hyponatremia defined as serum sodium increase >8 meq/L at 24 h (81). The risk of rapid correction was associated with younger age, female sex, schizophrenia, low Charlson comorbidity index, lower presentation [Na^+^] and low urine sodium concentration (<30 meq/L). In this study of 1,490 patients, 0.6% were found to have radiologic evidence of ODS that was noted mainly in subjects with beer potomania and hypokalemia with >8 meq/L rise in [Na^+^].

## Conclusions

Hyponatremia may be that clinical condition which induces the most iatrogenic harm when the diagnosis or the treatment is wrongly exercised. As discussed in this review, chronic hyponatremia represents a wrong turn that the body takes for a wide variety of reasons. This chronic hyponatremia must induce decreases in brain organic osmolytes lest the patient's brain swell and herniate, a major problem with acute hyponatremia. However, this depletion of organic osmolytes with chronic hyponatremia makes the patient susceptible to demyelination should correction of the hyponatremia be too aggressive. It is critical that acute and chronic hyponatremia be distinguished. Acute hyponatremia with attendant brain swelling must be corrected urgently. Chronic hyponatremia must be corrected at a rate consistent with brain organic osmolyte regulation. To manage hyponatremic patients appropriately, it is critical that physicians actively manage electrolytes over the first 48-h until the patient has regained osmolar stability.

## Author Contributions

MK wrote the section on acute hyponatremia. IO wrote the section on chronic hyponatremia. JS wrote the section on osmolytes, made the table, and the two figures. ZK wrote the therapy section, introduction, and the conclusions. All authors contributed to the article and approved the submitted version.

## Conflict of Interest

The authors declare that the research was conducted in the absence of any commercial or financial relationships that could be construed as a potential conflict of interest. The handling editor declared a past-co-authorship with JS and ZK.

## Publisher's Note

All claims expressed in this article are solely those of the authors and do not necessarily represent those of their affiliated organizations, or those of the publisher, the editors and the reviewers. Any product that may be evaluated in this article, or claim that may be made by its manufacturer, is not guaranteed or endorsed by the publisher.
